# Effect of an Integrated Exercise Training Program on Muscle Mass and Functional Capacity in Sarcopenic Patients With Decompensated Liver Cirrhosis

**DOI:** 10.7759/cureus.112441

**Published:** 2026-07-11

**Authors:** Rushikesh S Patil, Poovishnu T Devi, Kiran S Dhaygude, Janhavee S Brahmadande

**Affiliations:** 1 Cardiopumonary Sciences, Krishna College of Physiotherapy, Krishna Vishwa Vidyapeeth, Deemed to Be University, Karad, IND; 2 Cardiopulmonary Sciences, Krishna College of Physiotherapy, Krishna Vishwa Vidyapeeth, Deemed to Be University, Karad, IND

**Keywords:** bioelectrical impedance analysis, functional capacity, liver cirrhosis, muscle mass, sarcopenia, six-minute walk test

## Abstract

Background: Liver cirrhosis is an end-stage disease with extensive fibrosis that disrupts normal architecture and impairs hepatic function. Progression to decompensation triggers severe complications like ascites, jaundice, and hepatic encephalopathy. A major extrahepatic consequence is sarcopenia (muscle wasting), which affects over 65% of these patients. Driven by ammonia accumulation and chronic inflammation, this muscle loss drastically increases frailty and mortality risk. Combined aerobic and resistance exercise (CARE) represents a highly promising, underutilized rehabilitation strategy to restore their functional capacity.

Objective: The aim of this study was to assess how a structured, home-based, integrated exercise program influences muscle mass and physical functional capacity in sarcopenic patients with decompensated cirrhosis. It was hypothesized that CARE would yield measurable gains in skeletal muscle volume, exercise endurance, and liver-related biochemical parameters relative to standard care alone.

Methods: A randomized clinical trial at Krishna College of Physiotherapy, Karad, India, evaluated 40 participants aged 30-50 years with decompensated cirrhosis and a Child-Pugh score of 7-15. Simple random sampling allocated patients equally into a control group receiving standard care (n=20) or an experimental group receiving standard care plus a 12-week CARE program (n=20). The cohort comprised 24 males and 16 females, with etiologies including alcohol-associated liver disease (52.5%), hepatitis B (7.5%), and hepatitis C (5.0%). Functional capacity and muscle mass were assessed pre- and post intervention using the Six-Minute Walk Test (6MWT) and Bioelectrical Impedance Analysis (BIA), respectively. Statistical analyses utilized paired t-tests and Wilcoxon signed-rank tests, with significance set at p < 0.05.

Following the 12-week intervention, the experimental group demonstrated significant pre-to-post improvements across all metrics: 6MWT distance increased from 480.1±3.50 to 487.55±5.97 meters (p < 0.0001); serum glutamic-oxaloacetic transaminase (SGOT) dropped from 66.55±8.54 U/L to 47.3±12.9 U/L (p < 0.0001); serum glutamic-pyruvic transaminase (SGPT) decreased from 68.6±7.67 U/L to 54.15±10.03 U/L (p < 0.0001); and skeletal muscle mass increased from 24.16±2.25 to 24.77±2.96 kg (p = 0.0452). Between-group comparisons at post-intervention revealed that the experimental group achieved significantly greater improvements than the control group in 6MWT distance (p = 0.0055), SGOT (p = 0.0001), and SGPT (p = 0.0001). However, the post-intervention difference in muscle mass between the experimental and control groups did not reach statistical significance (p = 0.4134).

Conclusion: A structured, home-based CARE program produced significant improvements in functional capacity, biochemical markers, and muscle mass among sarcopenic individuals with decompensated cirrhosis. Exercise training is safe, feasible, and clinically beneficial in this population, offering a nonpharmacological strategy to mitigate sarcopenia and enhance quality of life. Incorporating rehabilitation into standard management of advanced liver disease may reduce morbidity, improve transplant outcomes, and extend survival. Future research should explore long-term adherence, optimal exercise intensity, and integration with nutritional interventions to maximize therapeutic benefit.

## Introduction

Liver cirrhosis is an end-stage liver disease characterized by extensive fibrosis and regenerative nodule formation that disrupt normal hepatic architecture and function. These structural changes impair hepatic blood flow and metabolic homeostasis, leading to progressive liver dysfunction. Decompensated cirrhosis develops when complications such as ascites, variceal bleeding, jaundice, or hepatic encephalopathy occur, reflecting portal hypertension and impaired liver function. Additional complications include edema, coagulopathy, spontaneous bacterial peritonitis, splenomegaly, sarcopenia, and renal dysfunction. Disease severity is commonly assessed using the Child-Pugh classification, with scores above 7 indicating decompensation [1-5].

The major causes of cirrhosis include chronic hepatitis B or C infection, excessive alcohol consumption, and metabolic dysfunction-associated steatotic liver disease (MASLD). Progressive hepatocellular injury and fibrosis eventually overwhelm the liver's ability to perform essential metabolic, synthetic, and detoxification functions. Chronic alcohol use is particularly associated with accelerated disease progression and decompensation [6, 7].

Sarcopenia, a common complication affecting over two-thirds of patients with decompensated cirrhosis, results from hyperammonemia, inflammation, hormonal disturbances, malnutrition, and altered protein metabolism. Elevated ammonia promotes oxidative stress, mitochondrial dysfunction, autophagy, and myostatin activation, leading to reduced muscle protein synthesis and increased muscle breakdown. Clinically, sarcopenia is characterized by loss of muscle mass, strength, and physical performance [8-12].

Studies demonstrate an inverse relationship between MASLD and skeletal muscle health, while concurrent loss of muscle and bone mass is increasingly recognized in chronic liver disease. Exercise, particularly progressive resistance training, is an effective non-pharmacological intervention for improving muscle mass, strength, and physical function in patients with sarcopenia [13-19].

Chronic liver inflammation can also lead to hepatopulmonary syndrome, characterized by excessive nitric oxide-mediated pulmonary vasodilation, ventilation-perfusion mismatch, intrapulmonary shunting, and impaired oxygen diffusion. These abnormalities contribute to hypoxemia and reduced exercise capacity [20, 21].

Regular exercise is a safe and effective strategy for improving functional capacity in cirrhosis. Combined aerobic and resistance exercise (CARE) enhances cardiorespiratory fitness, muscle strength, and physical performance. A recent meta-analysis reported an average improvement of approximately 49 meters in the Six-Minute Walk Test (6MWT) among patients participating in combined exercise programs. By targeting both cardiovascular and muscular impairments, CARE may help counteract sarcopenia, frailty, and exercise intolerance in clinically stable patients with decompensated cirrhosis [22,23].

This study aimed to evaluate the efficacy of a 12-week home-based CARE program in patients with decompensated liver cirrhosis and sarcopenia. The primary objective was to determine the impact of the CARE intervention on functional capacity, measured by the 6MWT. The secondary objectives were to evaluate structural muscle mass changes via Bioelectrical Impedance Analysis (BIA) and assess biochemical liver profiles (serum glutamic-oxaloacetic transaminase (SGOT)/serum glutamic-pyruvic transaminase (SGPT)). We hypothesized that the 12-week CARE protocol would yield significantly greater functional improvements compared to standard care while favorably modifying muscle mass and biochemical trajectories.

## Materials and methods

Study design 

This investigation was designed as a prospective, randomized, experimental clinical trial. Simple random sampling was employed to minimize selection bias and ensure each eligible participant had an equal chance of being allocated to either group, thereby controlling for potential confounders. All study activities took place at Krishna College of Physiotherapy, Karad, India, leveraging its dedicated clinical research facilities. A convenience sample of 40 participants (n=20 per arm) was targeted based on institutional recruitment feasibility and strict safety monitoring capacities within the study window.

Participants

A total of 40 participants were enrolled via simple random sampling. Inclusion required participants to be between 30 and 50 years of age, of either sex, with a confirmed diagnosis of decompensated liver cirrhosis and a Child-Pugh score in the range of 7 to 15 [24]. Individuals with coexisting cardiac conditions, coronary artery disease, arrhythmias, or dialysis-dependent chronic renal insufficiency were not eligible. All enrolled participants gave written informed consent before any data collection occurred. The study was conducted in accordance with the Declaration of Helsinki and received ethics committee approval from the institution. The Institutional Ethics Committee gave permission to initiate the research project (protocol number 289/2025-2026) on 5 November 2025.

Randomization and blinding

Participants were randomly assigned in a 1:1 ratio to either the CARE intervention group or the standard care control group. Sequence generation was performed by an independent biostatistician using a computer-generated random number schedule. To ensure strict allocation concealment, group assignments were housed in sequentially numbered, opaque, sealed envelopes that were opened only after baseline clinical testing was completed.

Due to the active behavioral nature of the home-based physical exercise intervention, blinding of the participants and the training instructors was not feasible (introducing a risk of performance bias). However, to minimize detection bias, all clinical outcome assessors responsible for conducting the 6MWT, BIA, and processing biochemical liver markers (SGOT/SGPT) were completely blinded to the treatment allocations throughout the entire 12-week study framework.

**Figure 1:**
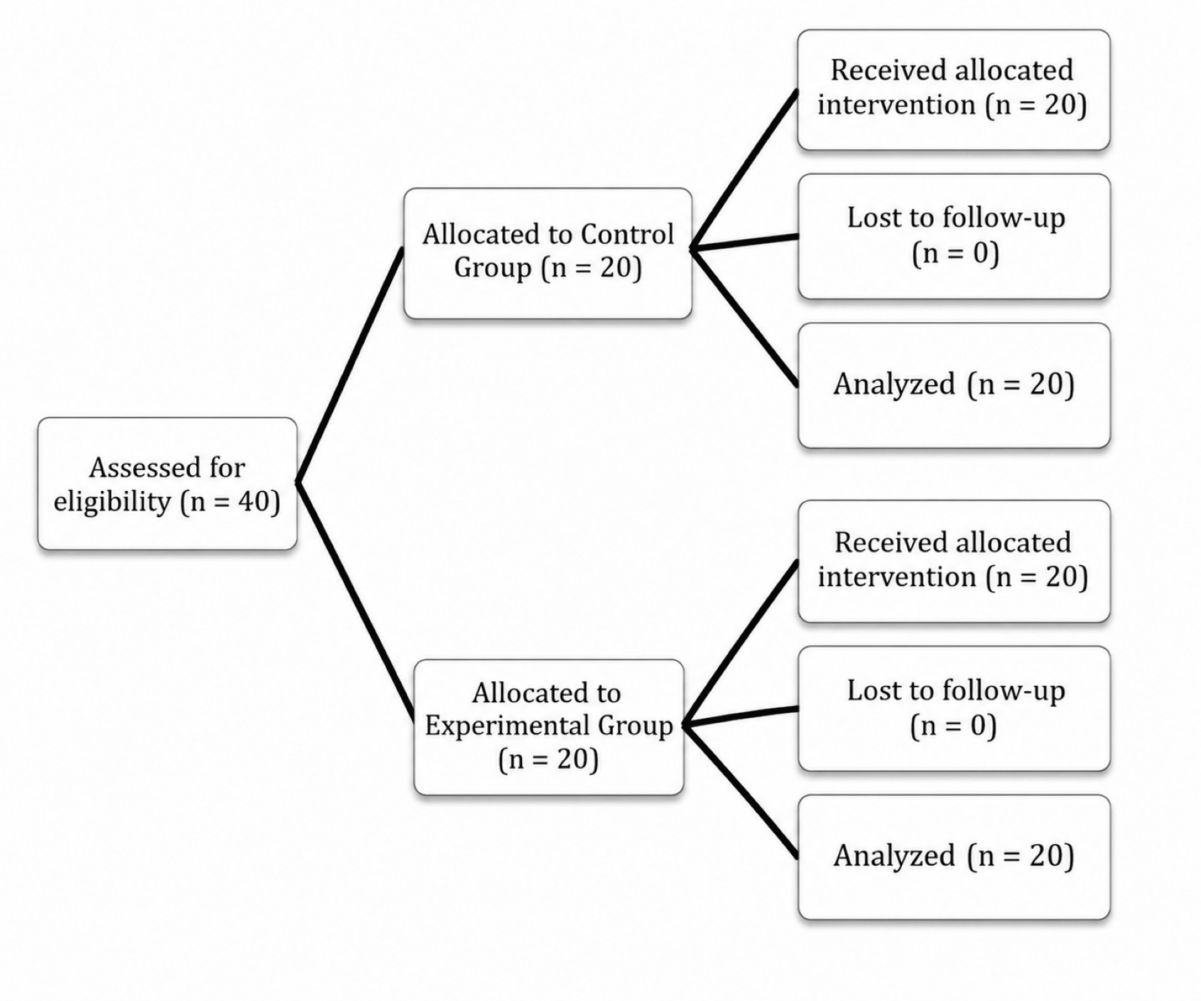
CONSORT flow diagram showing participant enrollment, randomization, allocation, follow-up, and analysis. CONSORT: Consolidated Standards of Reporting Trials

Participants were recruited using consecutive sampling, whereby all patients presenting to the clinic who met the inclusion criteria were sequentially screened and invited to participate until the target sample size of 40 was achieved. Following enrollment and baseline testing, simple random allocation was utilized to distribute the 40 participants into either the control group (n=20) or the experimental group (n=20) using a computer-generated randomization sequence to ensure an equal and unbiased distribution between the arms. Standard care for both the control and experimental groups was managed independently by the treating clinical hepatology team and was not dictated by the study protocol. This routine care was tailored to individual clinical needs and included standard pharmacological management (e.g., diuretics for ascites, lactulose or rifaximin for hepatic encephalopathy), routine medical follow-ups, and general dietary recommendations regarding protein and sodium intake. Because these treatments were managed dynamically based on clinical progression and were not rigidly standardized by the study protocol, this variation is acknowledged as a limitation of the study. All participants received their allocated interventions and completed the study protocol. No participants were lost to follow-up or excluded from the final analysis. Consequently, data from all 40 participants were included in the statistical analysis, with 20 participants analyzed in each group, as given in Figure 1.

Outcome measures

The 6MWT

The 6MWT is a well-established functional evaluation that records the maximum distance an individual can cover across six minutes of walking at a self-selected, submaximal pace [25]. This metric reflects real-world functional exercise tolerance by measuring the combined performance of the musculoskeletal, respiratory, and cardiovascular systems without requiring the patient to reach maximum exertion. Consequently, it is well-suited for fragile clinical cohorts, such as those with decompensated cirrhosis, who cannot tolerate maximal stress testing due to underlying frailty, fatigue, or comorbidities. The tool demonstrates an intraclass correlation coefficient of 0.97 (95% CI: 0.90-0.99; P < 0.001) across consecutive tests on identical subjects, indicating exceptionally low measurement variance (approximately 18.9) and an excellent correlation coefficient (r = 0.98).

Bioelectrical Impedance Analysis

BIA provides a practical, non-invasive diagnostic tool to monitor changes in muscle volume in decompensated cirrhotic patients, particularly when serial, bedside tracking is required [26]. BIA calculates fat-free mass and skeletal muscle metrics by measuring the body's native resistance and reactance against a low-voltage electrical current; these operational standards are validated by the European Society for Clinical Nutrition and Metabolism [23]. Regarding concurrent validity, BIA-calculated skeletal muscle mass correlates significantly with computed tomography (CT)-derived skeletal muscle index (SMI) metrics at the L3 level, which remains the diagnostic gold standard for assessing cirrhosis-associated sarcopenia. Evaluations within cirrhotic cohorts show a powerful correlation between BIA and CT tracking, ranging from r = 0.70 to 0.89 (P < 0.001), confirming strong diagnostic agreement between the two assessment methods.

Procedure

The trial followed a methodical, pre-specified protocol (Table 1) [17]. Ethics clearance from the institutional review committee was obtained at the outset to ensure full regulatory compliance. Recruitment proceeded according to defined inclusion and exclusion criteria to establish a clinically homogeneous sample. After enrolment, participants were randomized into two arms: Group A received standard medical care (control), and Group B underwent the structured exercise intervention (experimental). All participants completed baseline assessments before the intervention and identical post-intervention evaluations at the end of the study period. The resulting data were entered into statistical software, analyzed, and interpreted to assess the therapeutic value of the exercise program.

**Table 1:** Combined aerobic and resistance exercise (CARE) program protocol Reference: [17]

Parameter	Protocol Specification
Frequency	Three days per week on alternating days
Time	45 to 60 minutes total (includes 5–10 minute warm-up and 5–10 minute cool-down).
Aerobic Exercise Type	Structured brisk walking, stationary cycling, or low-impact aerobic stepping.
Resistance Exercise Type	Bodyweight and light resistance band exercises targeting major muscle groups (seated chest presses, bicep curls, wall squats, and calf raises).
Intensity Prescription	Aerobic: Light-to-moderate, Borg Rating of Perceived Exertion (RPE) of 11–13, or 40%–60% heart rate reserve. Resistance: one to two sets of 10–15 repetitions at a Borg RPE of 12–14.
Progression Criteria	Evaluated bi-weekly; if a participant completed two consecutive weeks with an RPE < 11, resistance was increased (higher band tension or +1 set), and aerobic duration was extended by 5 minutes (maximum 30 minutes continuous).

Supervision and Adherence Monitoring

The program was fully home-based but partially supervised via tele-rehabilitation. Physical therapists conducted weekly phone calls to review mandatory daily paper exercise logs maintained by the participants and to adjust progression parameters. Adherence was calculated as the percentage of prescribed sessions completed, with 80% completion deemed compliant.

Safety Monitoring & Stopping Criteria

Participants were strictly instructed to halt exercising immediately if they experienced red-flag symptoms, including sudden dizziness, severe dyspnea (Borg Dyspnea Scale >4), chest pain, claudication, profound exhaustion, or confusion/disorientation (suggestive of hepatic encephalopathy). Clinical worsening, such as an abrupt increase in abdominal girth (ascites) or lower limb edema, required temporary cessation of the programme and immediate referral to the primary hepatology team for clinical clearance.

Statistical analysis

Statistical analyses were performed using IBM SPSS Statistics software, version 25.0 (IBM Corp., Armonk, NY, USA). Data normality was evaluated using the Shapiro-Wilk test. Continuous variables are expressed as mean ± standard deviation (SD) for normally distributed data, or as median (interquartile range (IQR)) for non-normally distributed data.

Within-Group Pre-post Comparisons

Changes within each group from baseline to 12 weeks were analyzed using the paired t-test for normally distributed continuous variables (e.g., 6MWT distance) and the Wilcoxon signed-rank test for non-normally distributed variables.

Between-Group Baseline Comparisons

To confirm baseline comparability between the experimental and control groups, independent-samples unpaired t-tests were used for normally distributed continuous markers, the Mann-Whitney U test for non-normal continuous markers, and the chi-square test or Fisher's exact test for categorical variables (e.g., gender and etiology).

Between-Group Post-intervention Comparisons

The therapeutic superiority of the CARE intervention over standard care at 12 weeks was primarily evaluated using the independent-samples unpaired t-test (for normally distributed data, including post-intervention 6MWT and liver enzymes) and the Mann-Whitney U test (for non-normally distributed variables). Statistical significance was set a priori at p < 0.05 for all tests.

## Results

A total of 40 participants (100.0%) with decompensated liver cirrhosis were included in the study, revealing a mean age of 41.42±5.49 years with a majority composition of 24 males (60.0%) compared to 16 females (40.0%). In terms of etiology, alcohol-associated liver disease was the most common primary driver affecting 21 participants (52.5%), followed by minor viral representations of three participants (7.5%) for hepatitis B and two participants (5.0%) for hepatitis C. All participants maintained an overall baseline Child-Pugh score of 9.17±1.96, with an average baseline 6MWT distance of 478.5±4.45, mean SGOT levels of 66.93±7.22, mean SGPT levels of 68.20±7.21, and a mean muscle mass of 24.15±2.16, as presented in Table 2.

**Table 2:** Descriptive statistics of all participants ALD: alcohol-associated liver disease; HBV: hepatitis B virus; HCV: hepatitis C virus; CPS: Child-Pugh score; DLC: decompensated liver cirrhosis; 6MWT: Six-Minute Walk Test; SGOT: serum glutamic-oxaloacetic transaminase; SGPT: serum glutamic-pyruvic transaminase; MM: muscle mass; SD: standard deviation; n: number of participants.

Variable	Mean (SD)/Frequency (%)
Age, Mean (SD)	41.42 ± 5.49
Sex, n (%)	
Female	16 (40.0)
Male	24 (60.0)
ALD, n (%)	
No	19 (47.5)
Yes	21 (52.5)
Hepatitis B, n (%)	
No	37 (92.5)
Yes	3 (7.5)
Hepatitis C, n (%)	
No	38 (95.0)
Yes	2 (5.0)
Child–Pugh Score, Mean (SD)	9.17 ± 1.96
DLC, n (%)	
Yes	40 (100.0)
No	0 (0.0)
6MWT, Mean (SD)	478.5 ± 4.45
SGOT, Mean (SD)	66.93 ± 7.22
SGPT, Mean (SD)	68.20 ± 7.21
Muscle Mass, Mean (SD)	24.15 ± 2.16

At baseline, 40 participants were randomized equally between the control group (n = 20, 50.0%) and the experimental group (n = 20, 50.0%). Gender allocation differed slightly between the cohorts, with the control group containing 14 males (70.0%) and six females (30.0%), while the experimental group consisted of 12 males (60.0%) and eight females (40.0%). Alcohol-associated liver disease was closely balanced, impacting 11 control participants (55.0%) and 10 experimental participants (50.0%), whereas hepatitis B was present in one control individual (5.0%) and two experimental individuals (10.0%). However, a significant difference was noted in the distribution of hepatitis C (p < 0.0001), showing an asymmetrical allocation for this sub-metric. Both arms exhibited a 100% prevalence rate for decompensated liver disease, confirming identical primary diagnoses. The experimental cohort showed a slightly higher baseline 6MWT score than the control group (p = 0.0233), pointing to a slightly better initial walking capacity. No notable variations were observed between the cohorts regarding initial SGOT, SGPT, or muscle mass values, confirming matching hepatic enzyme and body composition profiles before the intervention, as presented in Table 3.

**Table 3:** Comparison of baseline (pre-test) descriptive statistics between control and experimental group. t value: independent t test; ALD: alcohol-associated liver disease; HBV: hepatitis B virus; HCV: hepatitis C virus; DLC: decompensated liver cirrhosis; 6MWT: Six-Minute Walk Test; SGOT: serum glutamic-oxaloacetic transaminase; SGPT: serum glutamic-pyruvic transaminase; SD: standard deviation; n: number of participants. *: This indicates a statistically significant difference in how hepatitis C was distributed between the control and experimental groups at baseline, whereas other variables matched.

Variables	Control (n=20)	Experimental (n= 20)	t value	p-value
Age, Mean (SD)	41.05±5.55	41.8±5.55	0.4270	0.6718
Sex, n%				
Female	6 (30)	8 (40)		
Male	14 (70)	12 (60)	0.1099	0.7403
ALD, n%				
No	9 (45)	10 (50)		
Yes	11 (55)	10 (50)	0.1003	0.7515
Hepatitis B, n%				
No	19 (95)	18 (90)		
Yes	1 (5)	2 (10)	0.3604	0.5483
Hepatitis C, n%				
No	19 (95.0)	19 (95.0)		
Yes	1 (5.0)	1 (5.0)	29.192	<0.0001*
DCL, n%	20 (100)	20 (100)		
6MWT, Mean (SD)	476.95±4.81	480.1±3.50	2.364	0.0233
SGOT, Mean (SD)	67.3±5.56	66.55±8.54	0.3290	0.7440
SGPT, Mean (SD)	67.8±6.90	68.6±7.67	0.3465	0.7309
Muscle Mass, Mean (SD)	24.13±2.124	24.16±2.25	0.9657	0.4328

The control arm demonstrated a statistically significant change in 6MWT distance over the study timeframe, indicating a marginal improvement in functional walking capacity. A significant drop was also recorded in their SGOT enzyme levels, demonstrating minor variations in hepatic markers. Conversely, while post-test SGPT metrics shifted downward slightly, this change failed to reach statistical significance. Muscular mass tracking remained virtually stationary between the baseline and post-test reviews, showing no meaningful change. Ultimately, these records reveal that standard care generated highly selective, minor updates in basic physical execution and individual chemical markers within the control cohort, as given in Table 4.

**Table 4:** Comparison of pre-test and post-test data of the outcome measures of control group. t value: independent t test; 6MWT: Six-Minute Walk Test; SGOT: serum glutamic-oxaloacetic transaminase; SGPT: serum glutamic-pyruvic transaminase; SD: standard deviation *: This confirms that the control group achieved a statistically significant improvement in their functional walking capacity over the 12-week timeframe using standard care alone.

Variables	Pre-test	Post Test	Mean Difference	t value	p-value
6MWT, Mean (SD)	476.95±4.81	481.2±4.81	-4.25	6.285	<0.0001*
SGOT, Mean (SD)	67.3±5.56	66.5±5.91	0.8	3.760	0.0013
SGPT, Mean (SD)	67.8±6.90	66.7±7.70	1.1	2.046	0.0548
Muscle Mass, Mean (SD)	24.13±2.124	24.125±1.83	0.01	0.04751	0.9626

The experimental cohort achieved a pronounced improvement in 6MWT distances, showing a substantial boost in physical endurance following the training protocol. Simultaneously, both SGOT and SGPT concentrations dropped sharply, signaling a meaningful improvement in serum markers of liver function. Furthermore, skeletal muscle mass metrics increased from pre-to-post test, showing a positive change in overall body composition. The highly significant p-values observed across all evaluated parameters confirm that these variations were driven by the intervention rather than random chance. The integrated training programme delivered substantial improvements across functional performance, metabolic liver markers, and skeletal muscle mass, as presented in Table 5.

**Table 5:** Comparison of pre-test and post test data of the outcome measures of experimental group. t value: independent t test; 6MWT: Six-Minute Walk Test; SGOT: serum glutamic-oxaloacetic transaminase; SGPT: serum glutamic-pyruvic transaminase; SD: standard deviation *: This denotes a highly statistically significant increase in functional walking distance from 480.1 to 487.55 meters after the 12-week intervention. This confirms that the combined aerobic and resistance exercise (CARE) program successfully and reliably improved physical endurance and cardiorespiratory fitness in the experimental group. **: This marks a highly statistically significant reduction in serum SGPT concentrations, dropping sharply from 68.6 to 54.15. This statistically sound decrease proves that the integrated exercise regimen directly helped optimize and normalize metabolic liver markers. ***: This indicates that the sharp drop in SGOT from 66.55 to 47.3 was a meaningful improvement driven by the training protocol rather than random chance and is highly significant.

Variables	Pre-test	Post Test	Mean difference	t value	p-value
6MWT, Mean (SD)	480.1±3.50	487.55±5.97	-7.4	10.856	<0.0001*
SGOT, Mean (SD)	66.55±8.54	47.3±12.9	19.25	12.189	<0.0001**
SGPT, Mean (SD)	68.6±7.67	54.15±10.03	14.45	12.066	<0.0001***
Muscle Mass, Mean (SD)	24.16±2.25	24.77±2.96	-0.61	2.144	0.0452

Post-intervention comparisons demonstrated that the experimental cohort achieved significantly longer distances on the 6MWT compared to the control group. Furthermore, post-treatment SGOT and SGPT concentrations were markedly lower in the experimental arm, confirming a more robust normalization of liver enzymes following the exercise protocol. The highly significant P-values achieved for the 6MWT, SGOT, and SGPT metrics confirm the clear advantages of the experimental training program over standard care. Although the experimental group displayed an upward trend in overall muscle mass, the final variance between the two groups did not reach statistical significance. The experimental exercise program outperformed standard care in boosting functional capacity and optimizing hepatic biochemical markers, as given in Table 6.

**Table 6:** Comparison of post-intervention values between the control and experimental groups t value: independent t test; 6MWT: Six-Minute Walk Test; SGOT: serum glutamic-oxaloacetic transaminase; SGPT: serum glutamic-pyruvic transaminase; SD: standard deviation; CI: confidence interval *: This indicates a statistically significant, superior reduction in SGOT levels for the experimental group (47.3) compared to the control group (66.5). This proves that the combined aerobic and resistance exercise (CARE) program was reliably more effective than standard medical care alone at normalizing this liver enzyme. **: This proves that the experimental exercise program yielded a statistically significant, superior normalization of this specific liver enzyme when compared directly against standard care at the end of the 12 weeks.

Variables	Post-intervention mean		Result	95%CI of Mean Diff		t value	p-value
	Control	Experimental		lower	Upper		
6MWT, Mean (SD)	481.2±4.81	487.55±5.97	-6.35	-10.86	-1.84	2.947	0.0055
SGOT, Mean (SD)	66.5±5.91	47.3±12.9	19.2	12.55	25.85	6.039	0.0001*
SGPT, Mean (SD)	66.7±7.70	54.15±10.03	12.55	6.63	18.47	4.4335	0.0001**
Muscle Mass, Mean (SD)	24.125±1.83	24.77±2.96	-0.64	-2.26	0.98	0.8270	0.4134

## Discussion

The present trial investigated whether a structured, home-delivered integrated exercise program could improve muscle mass and functional performance in sarcopenic patients with decompensated cirrhosis. Sarcopenia is now well-established as a major extrahepatic complication of chronic liver disease and independently forecasts adverse outcomes, including increased morbidity, frailty, repeat hospital admissions, inferior post-transplant results, and shortened survival. Despite advances in pharmacological management, rehabilitation-based interventions continue to be underemployed in this patient group.

From a pathophysiological standpoint, sarcopenia in cirrhosis results from converging biological disruptions such as systemic low-grade inflammation, ammonia overload, hormonal dysregulation, depleted hepatic glycogen reserves, heightened protein catabolism, mitochondrial impairment, nutritional deficiency, and physical deconditioning [11]. Patients in the decompensated phase experience profound fatigue and intolerance to physical effort, which compounds skeletal muscle atrophy and promotes increasingly sedentary behavior: a self-reinforcing cycle of physical deterioration. Therapeutic strategies capable of interrupting this vicious cycle carry considerable clinical potential [27].

The findings of this study are discussed according to the primary outcomes, namely functional capacity, skeletal muscle mass, and liver biochemical markers. These outcomes are interpreted alongside existing clinical evidence and physiological pathways to clarify the therapeutic role of exercise training in advanced liver disease.

This study confirms that a 12-week integrated exercise intervention produces significant improvements in aerobic capacity and skeletal muscle volume compared to baseline values and conventional care. These findings reinforce the perspective that sarcopenia represents a modifiable complication in advanced liver disease rather than an irreversible consequence.

Sarcopenia is established as an important prognostic metric in chronic liver diseases, showing direct ties to frailty, compromised physical performance, elevated rehospitalization rates, diminished quality of life, and accelerated mortality. Dasarathy previously noted that muscle loss in this population stems from a complex interaction of systemic hypermetabolism, altered hormonal axes, chronic inflammation, nutritional deficits, and blunted protein synthesis pathways [8]. Similarly, Zhou et al. improved on this by emphasizing the prognostic value of sarcopenia beyond simple age-related muscle loss, highlighting its role in predicting survival outcomes for liver transplant candidates [28]. Their assessments revealed that a low skeletal muscle index severely impacts survival probability during decompensated phases. Corrêa et al. noted that sarcopenia significantly increases mortality rates for patients on transplant waitlists, particularly among female candidates, even when adjusting for matched Model for End-Stage Liver Disease (MELD) scores [22]. These observations confirm that muscle wasting is a major driver of poor clinical outcomes rather than a passive byproduct of liver failure [28].

The significant improvement observed in functional capacity, as assessed using the 6MWT, represents one of the major findings of the present study. Participants in the experimental group demonstrated a statistically significant increase in walking distance following the intervention. Baseline 6MWT values improved from 480.1±3.50 meters to 487.55±5.97 meters after 12 weeks of training, with a mean improvement of approximately 7.4 meters (p < 0.0001). Although the control group also demonstrated mild improvement, the magnitude of change was substantially lower, and post-intervention comparison between groups revealed statistically significant superiority of the intervention program [29].

This improvement in aerobic performance can be attributed to several physiological adaptations driven by concurrent aerobic and resistance training. Cirrhotic patients experience restricted exercise tolerance due to impaired skeletal muscle oxygen extraction, mitochondrial dysfunction, autonomic instability, hepatopulmonary vascular changes, and muscle atrophy. Structured exercise training improves blood flow to peripheral muscles, enhances mitochondrial oxidative enzyme capacity, and increases capillary density, allowing for more efficient oxygen delivery and utilization during physical exertion. Krüger et al. observed that consistent physical training optimizes muscular oxidative pathways and mitigates inflammatory myopathies [30], while Green et al. demonstrated that regular exercise supports endothelial health and cardiovascular adaptation in chronic conditions [31].

These results align with previous studies assessing exercise interventions in liver disease cohorts. Zenith et al. reported significant increases in peak oxygen consumption (VO₂ peak), improved muscle volume, and lower fatigue scores after an 8-week supervised exercise trial in compensated cirrhotic patients [32]. Similarly, Berzigotti et al. reported that structured physical regimens optimized functional metrics, metabolic regulation, and muscle strength [33]. Compared to data published by Alameri et al., which showed a mean 6MWT improvement of 3.15 meters, this trial achieved larger functional gains [34]. This difference may stem from the integrated structure of our protocol, which combines aerobic and resistance training to simultaneously target cardiovascular endurance, muscular efficiency, and neuromuscular control.

Crucially, the improvement in 6MWT distance achieved in this trial exceeded the minimal clinically important difference reported for chronic liver disease cohorts, indicating that the change is both statistically valid and clinically meaningful. Enhanced walking tolerance reflects a better capacity for independent activities of daily living, which can help reduce physical disability and frailty in advanced cirrhosis.

The use of the 6MWT as a primary endpoint supports the methodological validity of these findings. This assessment has demonstrated high reliability and validity across cardiopulmonary, geriatric, and musculoskeletal research. Enright and Sherrill established robust normative profiles for healthy adults [35], while Guyatt et al. validated the protocol as a dependable index of submaximal functional exercise capacity. Its reliance on movements that mimic daily activities makes it appropriate for evaluating functional limitations in sarcopenic patients with advanced liver disease [36].

Regarding body composition, the experimental group demonstrated an encouraging within-group increase in skeletal muscle mass over the 12-week intervention period (24.16±2.25 to 24.77±2.96 kg, p = 0.0452). However, the post-intervention between-group comparison did not achieve statistical significance (p = 0.4134), meaning a robust therapeutic effect of the CARE program on muscle volume relative to standard care cannot be definitively concluded. This lack of between-group significance indicates that while a 12-week structured exercise regimen initiates a positive internal trajectory toward muscle preservation, it is insufficient to fully reverse sarcopenia in this short timeframe. The physiological response of muscle hypertrophy in decompensated cirrhosis is highly constrained by underlying metabolic stressors. Therefore, future protocols may require a longer intervention duration combined with targeted nutritional and amino acid optimization to translate these encouraging within-group trends into robust, statistically significant between-group muscle mass gains.

This increase in muscle mass is supported by known physiological pathways. Muscle wasting in liver disease is driven by accelerated protein breakdown, blunted anabolic signaling, hyperammonemia, chronic inflammation, and nutrient deficiencies. Resistance training stimulates muscle protein synthesis by activating anabolic pathways, such as the mammalian target of rapamycin (mTOR), while aerobic exercise improves insulin sensitivity, mitochondrial efficiency, and metabolic homeostasis. The combination of both training modalities likely supported better skeletal muscle remodeling in this cohort [8]. Larsson et al. characterized sarcopenia as a progressive loss of muscle fiber volume and cross-sectional area linked to denervation, tissue atrophy, and altered reinnervation patterns [37]. Low muscle mass should not be viewed in isolation; muscle performance and metabolic quality must also be factored into the clinical picture. This study supports these models by showing parallel improvements in both structural muscle mass and functional performance.

Several limitations should be noted when interpreting these findings. The study period was relatively short, and long-term follow-up was not conducted to assess the durability of the improvements. The sample size may have also limited the statistical power to detect significant between-group differences for certain metrics. Additionally, daily nutritional intake and serum inflammatory biomarkers were not measured, which could have provided deeper insights into the mechanisms underlying the observed muscular adaptations. Additionally, standard care was managed dynamically by the primary hepatology team rather than being rigidly standardized by a fixed study protocol, which may introduce unmeasured confounding factors regarding pharmacological and nutritional variations between participants. The use of BIA is an inherent limitation in decompensated cirrhosis, as subclinical ascites or fluctuating fluid retention can distort lean mass measurements. Minor baseline variances occurred by chance in hepatitis C distribution and initial 6MWT scores, introducing potential confounding variables.

The strengths of this study lie in its randomized clinical trial design, which safely evaluated a novel, home-based CARE program within a fragile, decompensated liver cirrhosis population. Furthermore, the study utilized multidimensional objective metrics, including submaximal functional capacity via the 6MWT, biochemical liver markers, and body composition data, to comprehensively track therapeutic outcomes. Finally, the implementation of a structured tele-rehabilitation monitoring model using exercise logs and weekly phone calls demonstrated excellent feasibility, achieving high participant adherence without any adverse clinical events.

Future research should utilize larger, multi-center cohorts, longer intervention periods, and detailed nutritional and metabolic tracking. Further studies are also needed to evaluate the combined effects of structured exercise, targeted nutritional supplementation, and behavioral strategies to optimize clinical outcomes for sarcopenic patients with advanced liver disease.

## Conclusions

In conclusion, this randomized clinical trial, a structured 12-week home-based integrated exercise training program, yielded significant improvements in submaximal functional capacity (6MWT), biochemical markers of liver function (SGOT/SGPT), and within-group skeletal muscle mass among sarcopenic individuals with decompensated liver cirrhosis. These findings demonstrate that functional performance and certain metabolic parameters are modifiable through targeted, non-pharmacological physical rehabilitation in this fragile population. While these preliminary results suggest that the intervention is safe and feasible, the trial did not directly evaluate long-term adherence, quality of life, psychosocial functioning, hospitalization rates, or transplant and survival outcomes. Consequently, these broader systemic benefits remain potential clinical implications that require validation in future multi-center research incorporating longitudinal tracking and standardized nutritional interventions. The 12-week CARE program safely improved functional capacity and biochemical liver profiles, though non-significant between-group differences in muscle mass preclude definitive claims of sarcopenia reversal.
